# Autoimmune susceptibility gene *PTPN2* is required for clearance of adherent-invasive *Escherichia coli* by integrating bacterial uptake and lysosomal defence

**DOI:** 10.1136/gutjnl-2020-323636

**Published:** 2021-02-09

**Authors:** Marianne Rebecca Spalinger, Ali Shawki, Pritha Chatterjee, Vinicius Canale, Alina Santos, Anica Sayoc-Becerra, Michael Scharl, Michel L Tremblay, James Borneman, Declan F McCole

**Affiliations:** 1 Division of Biomedical Sciences, University of California Riverside School of Medicine, Riverside, California, USA; 2 Department of Gastroenterology and Hepatology, University Hospital Zürich, Zürich, Switzerland; 3 Department of Biochemistry, McGill University, Montreal, Quebec, Canada; 4 Goodman Cancer Research Centre, Rosalind and Morris Goodman Cancer Research Centre, Montreal, Quebec, Canada; 5 Department of Plant Pathology and Microbiology, University of California Riverside, Riverside, California, USA

**Keywords:** bacterial infection, cellular immunity, E. Coli, macrophages, mucosal immunology

## Abstract

**Objectives:**

Alterations in the intestinal microbiota are linked with a wide range of autoimmune and inflammatory conditions, including inflammatory bowel diseases (IBD), where pathobionts penetrate the intestinal barrier and promote inflammatory reactions. In patients with IBD, the ability of intestinal macrophages to efficiently clear invading pathogens is compromised resulting in increased bacterial translocation and excessive immune reactions. Here, we investigated how an IBD-associated loss-of-function variant in the protein tyrosine phosphatase non-receptor type 2 (*PTPN2*) gene, or loss of PTPN2 expression affected the ability of macrophages to respond to invading bacteria.

**Design:**

IBD patient-derived macrophages with wild-type (WT) *PTPN2* or carrying the IBD-associated *PTPN2* SNP, peritoneal macrophages from WT and constitutive PTPN2-knockout mice, as well as mice specifically lacking *PTPN2* in macrophages were infected with non-invasive K12 *Escherichia coli*, the human adherent-invasive *E. coli* (AIEC) *LF82*, or a novel mouse AIEC (*m*AIEC) strain.

**Results:**

Loss of PTPN2 severely compromises the ability of macrophages to clear invading bacteria. Specifically, loss of functional PTPN2 promoted pathobiont invasion/uptake into macrophages and intracellular survival/proliferation by three distinct mechanisms: Increased bacterial uptake was mediated by enhanced expression of carcinoembryonic antigen cellular adhesion molecule (CEACAM)1 and CEACAM6 in *PTPN2*-deficient cells, while reduced bacterial clearance resulted from defects in autophagy coupled with compromised lysosomal acidification. In vivo, mice lacking *PTPN2* in macrophages were more susceptible to *m*AIEC infection and *m*AIEC-induced disease.

**Conclusions:**

Our findings reveal a tripartite regulatory mechanism by which PTPN2 preserves macrophage antibacterial function, thus crucially contributing to host defence against invading bacteria.

**Significance of this study**

**What is already known on this subject?**

- Alterations in microbial composition are associated with several inflammatory disorders, including inflammatory bowel disease (IBD).
- Increased presence of adherent-invasive *Escherichia coli* (AIEC) occurs in IBD patients.
- Variants in protein tyrosine phosphatase non-receptor type 2 (PTPN2) are associated with increased risk to develop IBD and loss of PTPN2 affects immune responses.

**What are the new findings?**

- PTPN2 crucially affects the ability of macrophages to clear invading bacteria.
- Increased invasion and intracellular replication of AIEC occurs in PTPN2-deficient macrophages.
- PTPN2 restricts bacterial growth and invasion of macrophages by three distinct mechanisms in vivo: PTPN2 restricts expression of carcinoembryonic antigen cellular adhesion molecule proteins that promote bacterial entry; PTPN2 maintains autophagy of invading bacteria; PTPN2 preserves lysosomal acidification that is required for bacterial clearance.

**How might it impact on clinical practice in the foreseeable future?**

- Provides a mechanistic explanation for the altered microbial composition in patients ith IBD carrying PTPN2 variants.
- Our results indicate PTPN2 as a crucial factor for anti-bacterial defence, and thus, position PTPN2 as a potential therapeutic target in patients with AIEC overgrowth.

## Introduction

The human intestine is populated with over 1000 different bacterial species, which form a complex ecosystem that significantly impacts health.^1^ The vast majority of these bacteria are harmless or even beneficial commensals and do not pose danger to the host.^2^ Certain bacterial species, however, can cause diseases or contribute to the emergence of chronic inflammatory conditions, including inflammatory bowel disease (IBD),^3^ which comprises ulcerative colitis (UC) and Crohn’s disease (CD). UC and CD are associated with intestinal dysbiosis^3 4^—a term describing reduced overall bacterial diversity and overgrowth of pathogenic strains, including adherent-invasive *Escherichia coli* (AIEC) species.^4–6^ In contrast to their harmless, non-invasive counterparts, AIEC can penetrate the mucus layer, adhere to intestinal epithelial cells (IEC), and invade and survive in intestinal macrophages.^7 8^

In healthy individuals, overgrowth and invasion of pathogenic microbes is restricted by a thick mucus layer, a tight epithelial cell barrier,^9^ and immune cells in the lamina propria, and especially macrophages residing directly beneath IEC efficiently eliminate invading pathogens.^10 11^ These macrophages display an anti-inflammatory, highly phagocytic phenotype^12^ and remove cell debris, dying cells and harmless bacteria in an immune-silent manner.^13^ Nevertheless, in response to barrier defects and/or infiltration of invading pathogens, intestinal macrophages acquire an inflammatory phenotype, secrete large amounts of inflammatory cytokines and initiate full-blown immune responses.^11–13^ Once an infection is cleared, the anti-inflammatory setting is restored.^11 12^ However, when clearance of invading pathogens is disturbed, due to overgrowth of pathogenic bacteria or genetic factors that compromise the return to an immune silent state, exacerbated immune responses cause chronic inflammation, and drive the development of IBD and other intestinal disorders.^14^

Besides microbial/immunological factors, variants in over 200 genes have been associated with an enhanced risk to develop IBD.^15–17^ Among these, single nucleotide polymorphism (SNP) rs1893217 in the gene locus encoding protein tyrosine phosphatase non-receptor type 2 (*PTPN2*), not only contributes to enhanced risk of IBD, but also promotes other inflammatory diseases including rheumatoid arthritis, type 1 diabetes, and metabolic syndrome—diseases which are associated with alterations in the intestinal microbiota.^18 19^ PTPN2 (also known as T cell protein tyrosine phosphatase) loss, or presence of SNP rs1893217, results in aberrant T cell activation/differentiation,^20^ hyper-responsiveness to interferon (IFN)-γ and EGF,^21^ and defective autophagy—a process important for intracellular bacteria handling.^22^ In mice, full-body *Ptpn2*-knockout leads to severe systemic inflammation and death 3–5 weeks after birth.^23^ T cell-specific deletion promotes susceptibility to intestinal inflammation and alters the intestinal microbiome (depletion of potentially beneficial microbes, decreased bacterial richness, reduced levels of butyrate producers, increase of potential pathobionts^20^) similar to that observed in IBD patients carrying *PTPN2* SNP rs1893217.^24^ This demonstrates the importance of *PTPN2* and its potential role in controlling bacterial invasion. However, it is unclear how loss of PTPN2 affects the ability of macrophages to prevent bacterial infections.

We previously found that full-body *Ptpn2* knockout mice exhibit a significant expansion of a novel mouse AIEC (*m*AIEC).^25^ The aim of this study was to identify whether loss of PTPN2 activity compromises macrophage-mediated host defences against AIEC and to assess the mechanisms by which loss of PTPN2, or the presence of SNP rs1893217 SNP, compromises the ability of macrophages to clear AIEC infections.

## Methods

### Reagents

Sources of material and reagents used in this study are listed in online supplemental table S1.

10.1136/gutjnl-2020-323636.supp1 Supplementary data

### Patient-derived peripheral blood mononuclear cells

Whole blood samples were obtained from previously genotyped, sex and age-matched patients from the Swiss IBD cohort and healthy volunteers. All patients presented with quiescent disease at the time of sample collection. A list of patient characteristics and medication at the time of blood collection can be found in online supplemental table S2. All patients and healthy controls signed informed consent before study inclusion and the local ethics commission approved the study (Cantonal Ethics Commision Zurich, Switzerland; Approval number EK-1755).

### Mice

Heterozygous *Ptpn2* knockout (*Ptpn2*-Het) mice on a Balb/c background were obtained from Michel L. Tremblay at McGill University. *Ptpn2-*Het mice were bred with each other to obtain wild-type (WT), *Ptpn2*-Het and *Ptpn2*-KO littermates and cells obtained at an age of 3 weeks. Mice with a loxP flanked *Ptpn2* gene expressing Cre under the Lysozyme2 promoter (*Ptpn2*-LysMCre mice^26^) were bred with *Ptpn2^fl/fl^* mice to obtain *Ptpn2^fl/fl^* and *Ptpn2-*LysMCre littermates. Eight to ten weeks old littermates were used. For in vivo autophagy activation, mice were injected intraperitoneally daily with 1 mg/kg rapamycin in vehicle (4% ethanol, 5% polyethylene glycol, 5% tween 80) starting 1 day prior to the first bacterial gavage. Vehicle treated mice served as controls. For in vivo carcinoembryonic antigen cellular adhesion molecule (CEACAM)1 inhibition, mice were injected intraperitoneally daily with 10 mg/kg anti-CEACAM1 antibody (clone CC1) starting 1 day prior to the first bacterial gavage.

### Bacterial strains and infections

For experiments, K12 (ATCC), LF82^8^ and mouse AIEC (*m*AIEC)^27^ were cultured in Luria-Bertani (LB) medium overnight from frozen stocks, subcultured into fresh LB, harvested in the log-phase, washed in PBS, and macrophages infected at an MOI of 20. After 2 hours, macrophages were washed twice with Gentamycin (100 µg/mL)-containing culture medium and incubated in gentamycin (20 ug/mL)-containing culture medium. Unless otherwise stated, incubation times are given as the time of incubation after the Gentamycin washes. *m*AIEC expressing a plasmid containing the mCherry fluorescent protein were used for localisation studies.

### Macrophages

Macrophages were generated from human peripheral blood mononuclear cells, mouse bone marrow and THP-1 monocytes as detailed in online supplemental methods. Purity and differentiation was checked by flow cytometry and >96% of the cells were identified as macrophages. THP-1 cells with stable *PTPN2* knockdown using shRNA and transfected with WT *PTPN2*, variant *PTPN2* or an empty vector (EV) were obtained from Prof. Scharl at the University Hospital Zurich, University of Zurich, Zurich, Switzerland. Due to partially retained endogenous PTPN2 expression, variant expressing cells functionally resemble cells from heterozygous variant carriers.

### Flow cytometry

For flow cytometry, bone marrow derived macrophages (BMDM) or peritoneal macrophages were infected with *m*AIEC expressing a mCherry expressing plasmid or pHRhodo-K12 particles, detached from the culture plate using 2 mM ethylenediaminetetraacetic acid (EDTA) in PBS, washed twice and directly analysed on an LSRII flow cytometer. For measurement of reactive oxygen species (ROS), the cells were incubated with CellROX reagent (500 nM, Thermo Fisher Scientific) for 30 min at 37°C, washed in PBS and analysed immediately on an LSRII flow cytometer. For measurement of lysosomal acidification, the cells were incubated with LysoTracker green (25 nM, Thermo Fischer Scientific) for 1 hour, washed with PBS and analysed immediately on an LSRII flow cytometer.

### Immunofluorescent imaging, confocal microscopy

For immunofluorescence imaging, macrophages were seeded on cover slides after PMA pulsing and infected 24 hours later with *m*AIEC-mCherry or pHRhodo-K12 particles as described above. Staining for lysosome-associated membrane protein (LAMP)-1 and LC3B was performed according to standard procedures and as detailed in online supplemental methods.

### CEACAM1 and CEACAM6 inhibition in vitro

For blocking of CEACAMs, cells were incubated for 1 hour with 10 µg/mL antimouse CEACAM1 or antihuman CEACAM6 antibody prior to infection with K12, LF82 or *m*AIEC.

### siRNA treatment

STAT1 was silenced in peritoneal macrophages using a predesigned pool of 3 small-interfering RNAs (siRNA) from Dharmafect and Lipofectamine transfection reagent according to the manufacturer’s instructions, medium was replaced 16 hours after the transfection. Bacterial infections were carried out 48 hours later.

Protein and RNA extraction, Western Blotting and quantitative PCR was performed according to standard procedures and as described in online supplemental methods.

### PTPN2 activity assay

PTPN2 phosphatase activity was assessed as described previously^28^ using a phosphatase activity assay (Thermo Fisher Scientific). In brief, PTPN2 was immunoprecipitated from the lysates and samples dissolved in phosphatase-assay buffer, DiFMUP substrate added, and phosphatase activity measured every 10 min for 120 min to reliably determine phosphatase activity.^28^

### In vivo AIEC infection

Mice aged 8–10 weeks old were infected on four consecutive days with 10^9^ K12, LF82 or *m*AIEC/day. Disease activity was evaluated using the following parameters: weight loss, reduced grooming, reduced activity, stool consistency, overall appearance. Bacterial load was determined by homogenisation of faeces or tissues in 0.5 mL PBS and plating on LB agar.

### Quantification and statistical analysis

Data are represented as mean of n biological repetitions±SE of the mean. Data followed a Gaussian distribution and variation was similar between groups for conditions analysed together. Significant differences were determined using GraphPad Prism V.9 software using analysis of variance. P values below 0.05 were considered significant. Mice were randomly distributed into experimental groups after matching for age and gender. Numbers of replicates are given in the figure legends. Investigators were blinded for weight recording, evaluation of disease activity, and sample collection. No data points were excluded from statistical analysis.

## Results

### Loss of PTPN2 promotes AIEC uptake and survival in macrophages

**Figure 1:**
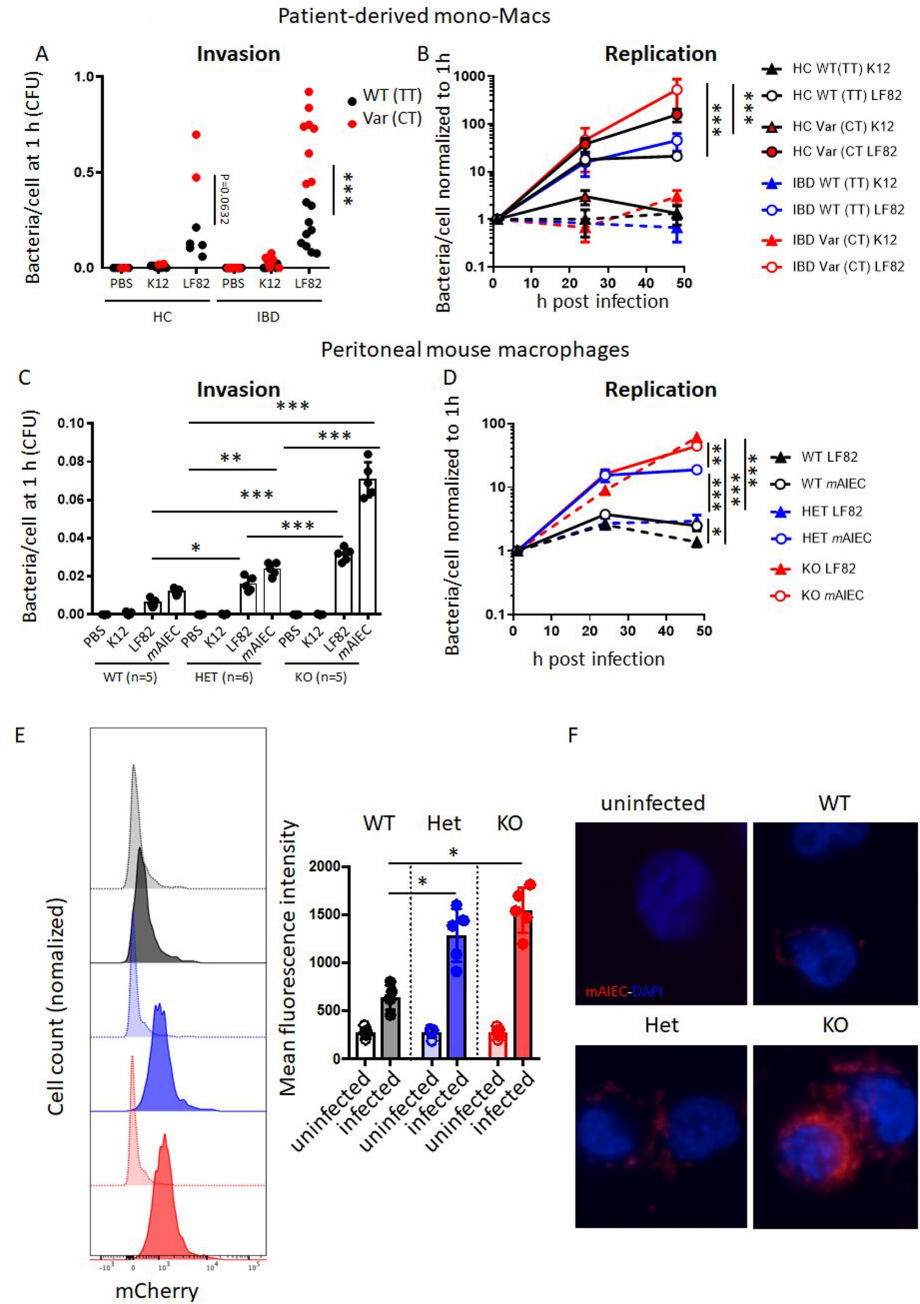
Loss of PTPN2 promotes uptake and replication of AIEC in macrophages. (A, B) Macrophages from healthy controls (HC), or patients with IBD homozygous for the wild-type (=C allele; WT (CC), HC: n=5, IBD: n=9) or heterozygous for the disease-associated (=T allele) variant (Var (CT), HC: n=2, IBD: n=8) in PTPN2 SNP rs1893217 were infected with non-invasive K12 *E. coli* or the AIEC strain LF82 for 2 hour, washed with PBS and incubated with gentamycin and macrophage uptake after 1 hour (A) and intracellular replication over time (B) analysed. (C, D) Peritoneal macrophages were isolated from WT, *Ptpn2-*Het (HET) or *Ptpn2*-KO (KO) mice and infected with K12, LF82 or a mouse AIEC (*m*AIEC) strain as described in A, B and analysed for bacterial uptake (C) and replication (D). (E, F) Bone marrow-derived macrophages were generated from WT, HET and KO mice, infected with mCherry-tagged *m*AIEC as in B and mCherry fluorescence measured after 3 hours by flow cytometry (E) and fluorescent imaging (F). Scale bar: 50 µm. Asterisks denote statistical significances (*P<0.05, **p<0.01, ***p<0.001, ANOVA with Bonferroni correction for multiple testing). See also online supplemental figure 1. AIEC, adherent-invasive *Escherichia coli*; ANOVA, analysis of variance; IBD, inflammatory bowel disease.

First, we assessed how loss of *PTPN2* or presence of the disease-associated variant in SNP rs1892317 in the gene locus encoding *PTPN2* affects bacterial uptake and intracellular replication in macrophages. Therefore, we employed *PTPN2*-silenced THP-1 monocytes that were stably transfected with lentiviral constructs containing normal (WT) *PTPN2*, an EV or the disease-associated *PTPN2* variant (Var; online supplemental figure S1A).^29^ These cells were exposed to non-invasive *E. coli* K12, the human LF82 AIEC, or a novel *m*AIEC recently discovered in our lab.^27^ EV-transfected (PTPN2-deficient) macrophages were highly susceptible to LF82 and *m*AIEC uptake, an effect mirrored in macrophages expressing the *PTPN2* loss-of-function variant (online supplemental figure 1A, B). Bacterial replication was increased in *PTPN2*-deficient and *PTPN2*-variant macrophages when compared with macrophages expressing the WT variant (online supplemental figure 1C). To confirm the effect of *PTPN2* SNP rs1893217, we used monocyte-derived macrophages from IBD patients from the Swiss IBD cohort previously genotyped as WT (TT) or heterozygous (CT) for SNP rs1892317. While uptake and replication in macrophages from *PTPN2* WT healthy control and IBD patients was similar, there was highly increased bacterial uptake and increased bacterial proliferation in macrophages from *PTPN2-*variant patients (figure 1A, B). Consistent with previous reports,^29^ presence of SNP rs1893217 reduced PTPN2 phosphatase activity, while protein expression levels were not affected (online supplemental figure 1D–G). We did not observe differences between macrophages from UC versus CD patients (data not shown).

In peritoneal macrophages from *Ptpn2*-het mice, uptake of LF82 and *m*AIEC was clearly increased, and further enhanced in homozygous *Ptpn2*-KO macrophages (figure 1C). Moreover, bacterial replication was elevated in *Ptpn2*-KO macrophages, an effect also partially visible in macrophages from *Ptpn2*-het mice (figure 1D). Increased bacterial uptake on loss of *Ptpn2* was confirmed by flow cytometry (figure 1E) and immunofluorescent imaging (figure 1F) in BMDM infected with mCherry-tagged *m*AIEC. Conversely, K12 uptake was very low (not shown). The inability of PTPN2-defective macrophages to clear intracellular bacteria was not limited to AIEC, but was also observed when macrophages were infected with the mouse enteropathogen *C. rodentium* (online supplemental figure 2). Taken together, this indicates that *Ptpn2*-deficient macrophages take up more bacteria than *Ptpn2*-competent macrophages, and that loss of *Ptpn2* compromises intracellular bacterial handling and elimination.

### Increased bacterial uptake is mediated via enhanced CEACAM6/CEACAM1 expression in *PTPN2*-deficient/defective macrophages

**Figure 2:**
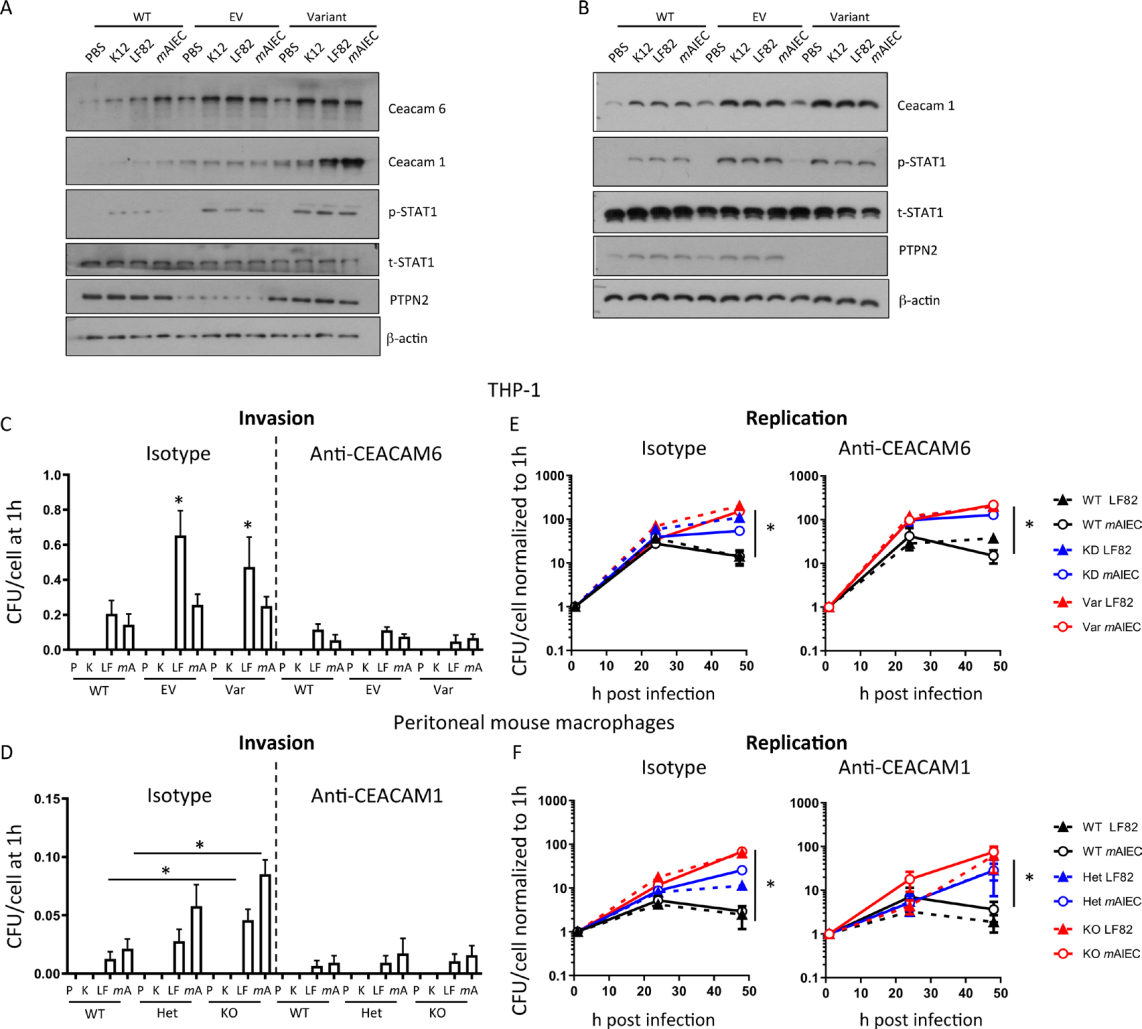
Loss of PTPN2 promotes CEACAM1 and CEACAM6 expression in macrophages. (A) PTPN2 was silenced in THP-1 macrophages prior to transfection with the major (WT) allele in SNP rs1893217, an empty vector (EV), or the minor (Var) allele in PTPN2. After selection of stable clones, cells were infected with K12, LF82 or *m*AIEC for 1 hour. Depicted are representative Western blot images of the indicated proteins. (B) Peritoneal macrophages were isolated from WT, *Ptpn2-*Het (HET) or *Ptpn2*-KO (KO) mice and infected with K12, LF82 or *m*AIEC for 1 hour. Depicted are representative Western blot images of the indicated proteins. (C–F) WT, EV and Var THP-1 cells (C, E) and peritoneal macrophages from WT, Het and KO mice (D, F) were infected with K12, LF82 or *m*AIEC for 2 hours, incubated with gentamycin and bacterial load (C, D) and replication (E, F) determined at the indicated time points. Asterisks denote statistical significances (*p<0.05, **p<0.01, ***p<0.001, ANOVA with Bonferroni correction for multiple testing). ANOVA, analysis of variance; *m*AIEC, mouse adherent-invasive *Escherichia coli*; PTPN2, protein tyrosine phosphatase non-receptor type 2; WT, wild-type.

LF82 uses CEACAM6 to adhere to and invade human IEC.^30^ To identify whether altered CEACAM expression contributes to increased AIEC uptake/invasion in *PTPN2*-deficient macrophages, we determined expression levels of CEACAM6 (human cells) and CEACAM1 (mouse cells; CEACAM6 is not expressed in mice).^31^ *PTPN2*-deficient and *PTPN2*-variant human THP-1 cells expressed elevated basal levels of CEACAM6 and CEACAM1, and AIEC further increased CEACAM1/6 expression (figure 2A). In addition, we observed enhanced levels of Signal-transducer and activator of transcription (STAT1) phosphorylation, which has been reported to promote CEACAM1, and to a lesser extent CEACAM6, expression^32^ (figure 2A). In mouse macrophages, we detected increased levels of CEACAM1 in *Ptpn2*-het and *Ptpn2*-KO macrophages and bacteria-induced STAT1 phosphorylation was increased (figure 2B). CEACAM1 (mouse) and CEACAM6 (human) inhibition with specific antibodies reduced bacterial invasion (figure 2C, D), indicating that AIEC entered the cells at least partially by attaching to CEACAM1/CEACAM6. In contrast, CEACAM inhibition did not affect intracellular bacterial replication, and even after CEACAM1/CEACAM6 inhibition, *PTPN2*-deficient and *PTPN2*-knockdown/variant THP-1 macrophages showed a faster increase in intracellular bacterial load (figure 2E, F), indicating that the increased replication was not due to increased bacterial uptake.

**Figure 3:**
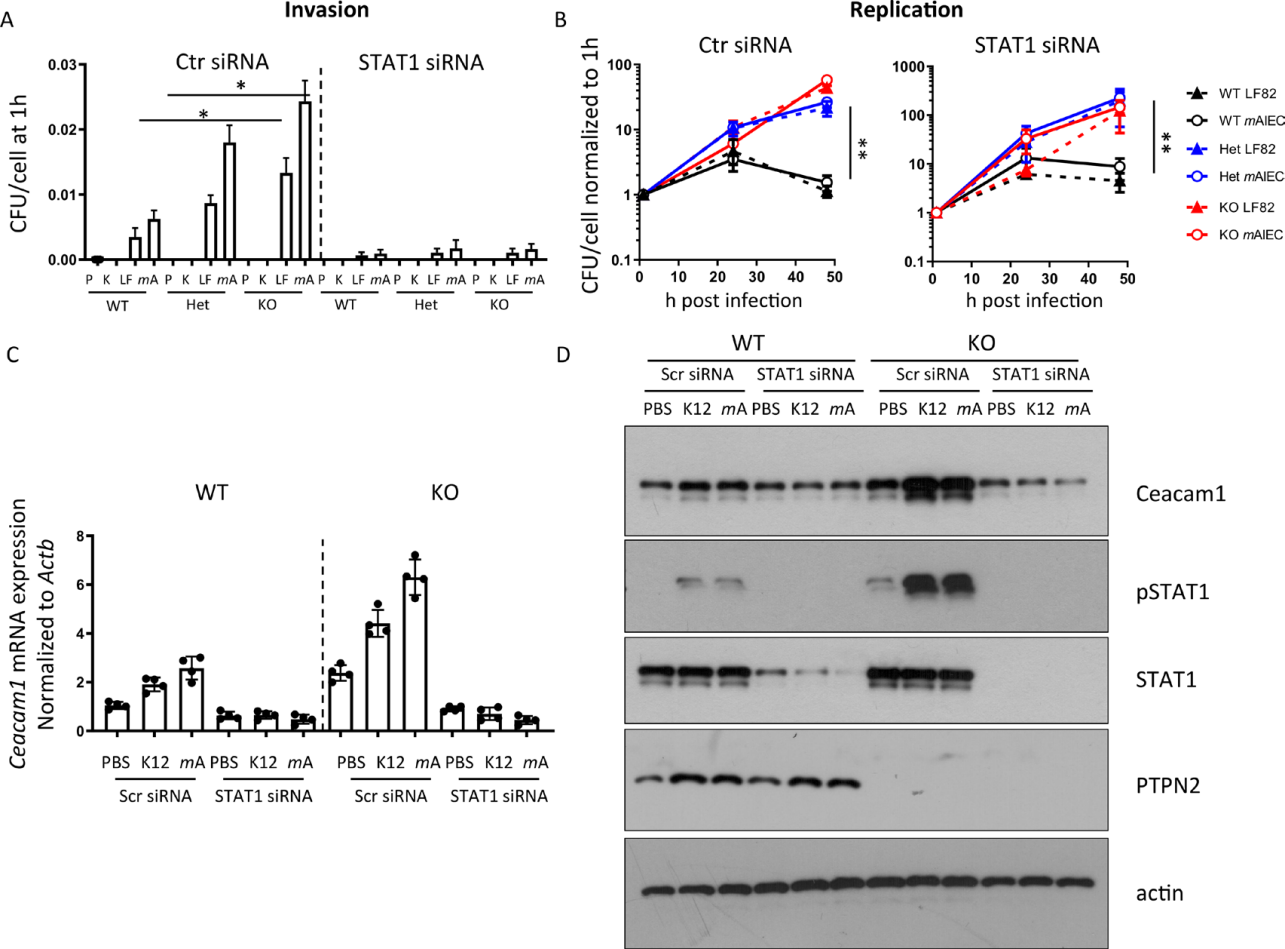
STAT1 inhibition normalises CEACAM expression and inhibits bacterial uptake. Peritoneal macrophages were isolated from WT, *Ptpn2-*Het (HET) or *Ptpn2*-KO (KO) mice, STAT1 silenced using siRNA and cells infected 24 hours later with K12, LF82 or *m*AIEC for 2 hours, incubated with gentamycin containing medium and (A) bacterial uptake and (B) replication analysed at the indicated time points; (C) mRNA expression of CEACAM1 normalised to untreated control and *Actb* after 24 hours; or (D) for the indicated proteins by Western blot after 30 min. Asterisks denote statistical significances (*p<0.05, **p<0.01, ANOVA with Bonferroni correction for multiple testing). ANOVA, analysis of variance; CEACAM, carcinoembryonic antigen cellular adhesion molecule; *m*AIEC, mouse adherent-invasive *Escherichia coli*; STAT1, signal-transducer and activator of transcription 1; WT, wild-type.

Given the increased levels of STAT1 phosphorylation and previous reports that IFN-γ-driven STAT1 activation promotes CEACAM1 and CEACAM6 expression,^32^ we assessed whether knockdown of STAT1 affected bacterial load. STAT1 siRNA-treatment decreased bacterial uptake (figure 3A) but did not affect bacterial replication (figure 3B). STAT1 silencing inhibited bacteria-induced CEACAM1 mRNA and protein induction (figure 3C, D), indicating that STAT1 mediates increased CEACAM1 expression in *Ptpn2*-KO macrophages.

### Increased bacterial survival is partially due to defects in autophagy

**Figure 4:**
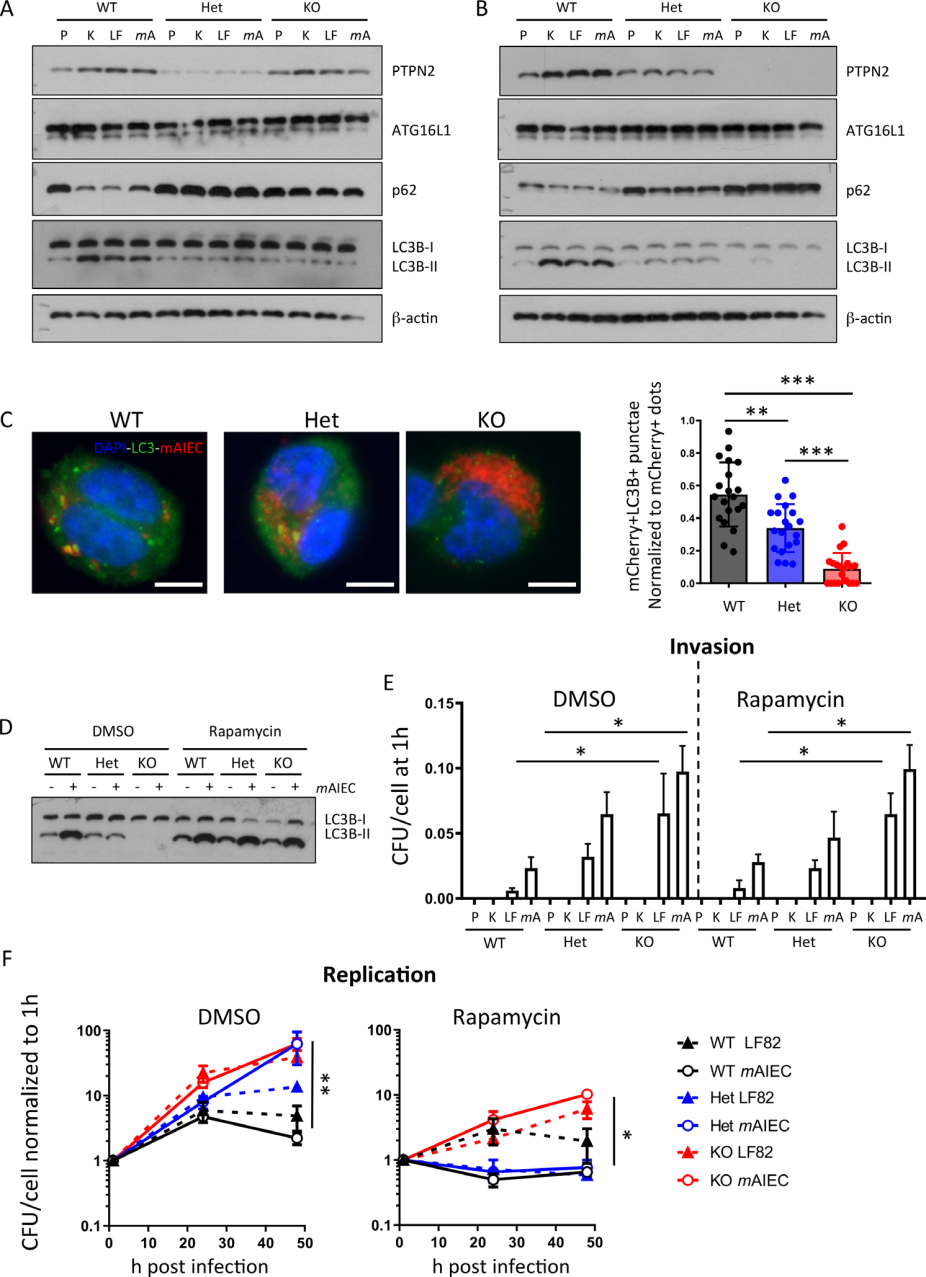
Defective autophagy in PTPN2-deficient cells contributes to enhanced bacterial replication. (A) *PTPN2* was silenced in THP-1 macrophages prior to transfection with WT PTPN2, an empty vector (EV), or Var *PTPN2* as in figure 1 and infected with K12, LF82 or B2 for 24 hours. Depicted are representative Western blots for the indicated proteins. (B) Peritoneal macrophages were isolated from WT, *Ptpn2-*Het (HET) or *Ptpn2*-KO (KO) mice and infected with K12, LF82 or *m*AIEC for 24 hours. Depicted are representative Western blot pictures for the indicated proteins. (C) Peritoneal macrophages were infected with mCherry expressing *m*AIEC for 4 hours and stained for LC3B. Bacteria colocalising with autophagosomes versus total bacteria were counted in 20 cells/condition. Scale bar: 10 µm. (D–F) Peritoneal macrophages were infected with K12, LF82 or *m*AIEC and incubated in the presence of rapamycin for the indicated time. (D) Representative Western blot picture for LC3B, (E) bacterial uptake after 1 hour, and (F) bacterial replication. (*P<0.05, **p<0.01, ***p<0.001). ANOVA, analysis of variance; *m*AIEC, mouse adherent-invasive *Escherichia coli*; PTPN2, protein tyrosine phosphatase non-receptor type 2; WT, wild-type.

Loss of *PTPN2* compromises autophagy in IEC and THP-1 monocytes.^29^ Since autophagy is an important factor for bacterial handling in the intestine,^33^ we investigated whether increased bacterial replication in *PTPN2*-deficient/variant macrophages might be due to defects in autophagy. In WT macrophages, infection with K12, LF82, or *m*AIEC resulted in enhanced conversion of LC3B into its lipidated form (LC3B-II) and a decrease of p62, indicating autophagy activation, while levels of ATG16L1 were not affected. *PTPN2*-deficient or variant THP-1 cells, however, failed to induce autophagy (figure 4A). Likewise, autophagy induction following bacterial infection was reduced in macrophages from *Ptpn2-*Het and completely absent in those from *Ptpn2*-KO mice (figure 4B). In WT and *Ptpn2-*Het macrophages, *m*AIEC colocalised with autophagosomes (visible as LC3B bright punctae that colocalise with mCherry-tagged *m*AIEC) although to a higher extent in WT than in *Ptpn2*-Het cells. In *Ptpn2*-KO cells, which exhibited a higher bacterial burden than WT cells, however, very few LC3B bright punctae were visible and they did not colocalise with mCherry-tagged *m*AIEC (figure 4C). This clearly indicates that *Ptpn2*-deficient and *PTPN2*-variant cells are defective in autophagy induction on bacterial infection. Autophagy induction via rapamycin induced autophagy in *Ptpn2-*Het and *Ptpn2*-KO macrophages (figure 4D), but had no effect on bacterial uptake (figure 4E), and only partially reduced bacterial replication (figure 4F). Conversely, autophagy inhibition using 3-methyladenine (3-MA) resulted in enhanced bacterial proliferation in WT macrophages, but had no effect on bacterial proliferation in PTPN2-deficient macrophages (online supplemental figure 3A). This indicates that loss of (functional) *Ptpn2* results in defective autophagy but the effect on bacterial replication seems only partially due to deficient autophagy. While autophagy was clearly reduced in PTPN2-deficient/variant macrophages, loss of (functional) PTPN2 had no effect on bacteria-induced ROS production (online supplemental figure 3B).

### *Ptpn2*-deficiency results in defective lysosomal acidification

**Figure 5:**
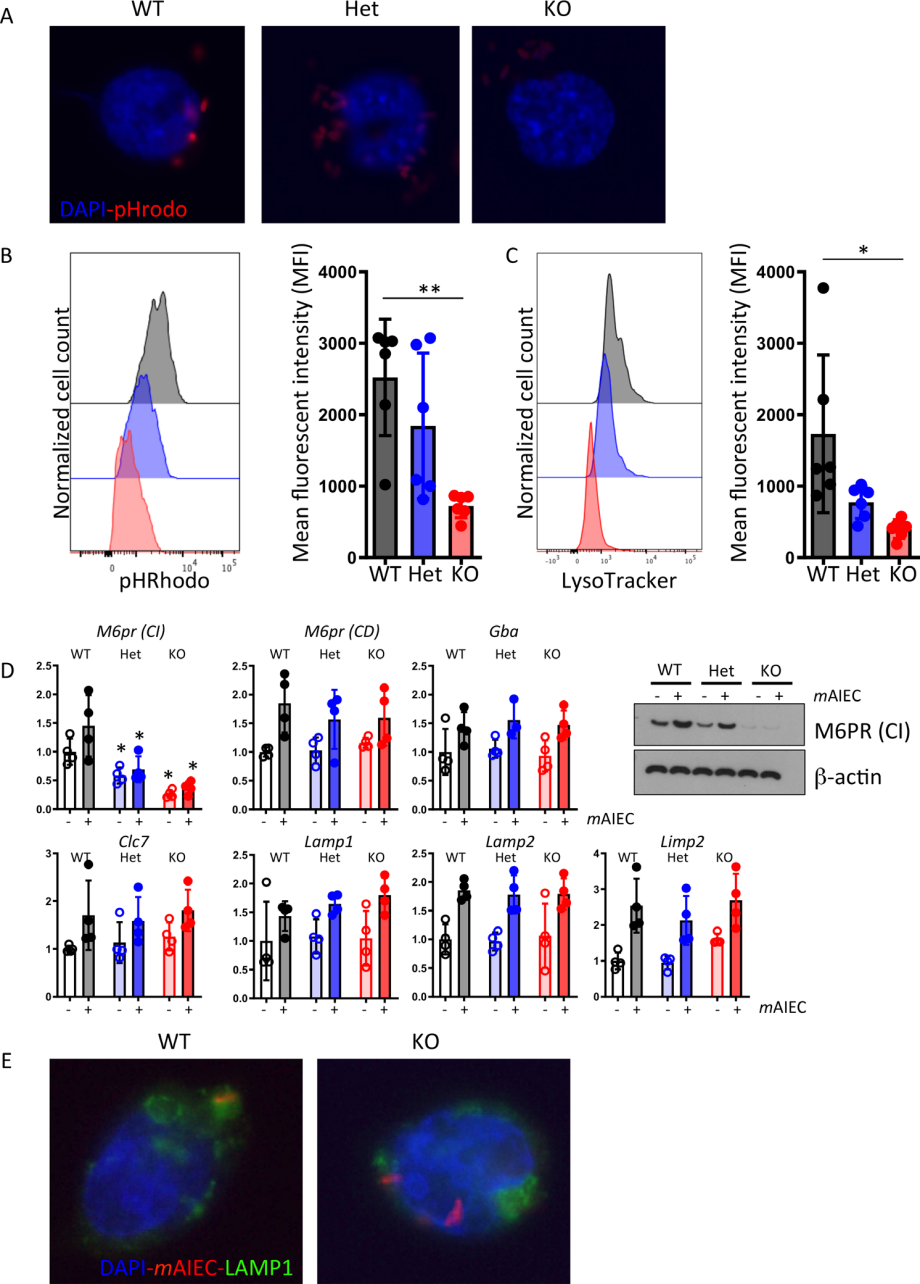
Disturbed lysosomal function in PTPN2-defective cells. (A, B) Peritoneal macrophages from WT, *Ptpn2-*Het (HET) or *Ptpn2*-KO (KO) mice were infected with K12-pHRhodamine particles that are fluorescent in acidic environment. Depicted are (A) representative images and (B) flow cytometry measurements of rhodamine fluorescence. (C) Peritoneal macrophages were infected with *m*AIEC and stained with LysoTracker and analysed by flow cytometry. (D, E) Peritoneal macrophages were infected with mCherry-expressing *m*AIEC. The graphs show (D) mRNA expression of the indicated genes and representative Western blot for cation-independent mannose-6-phosphate receptor protein after 24 hours, and (E) representative images from LAMP1 immunofluorescence. Scale bars: 10 µm. *p<0.05, **p<0.01, ANOVA. ANOVA, analysis of variance; LAMP1, lysosome-associated membrane protein; *m*AIEC, mouse adherent-invasive *Escherichia coli*; M6pr, mannose-6-phosphate receptor; PTPN2, protein tyrosine phosphatase non-receptor type 2; WT, wild-type.

Macrophages usually degrade phagocytozed dead cells and bacteria in lysosomes, a process promoted by autophagy and dependent on acidification of lysosomes.^34 35^ Since *Ptpn2*-defective macrophages were not able to degrade bacteria efficiently even when autophagy was restored, we next assessed whether this might be due to defective lysosomal acidification. For this aim, we used pHRhodamine-coupled bacterial particles, which show an increase in fluorescence in acidic environments. In WT macrophages, fluorescence was clearly visible indicating that the bacterial particles were transported into lysosomes with low pH (figure 5A). In *Ptpn2*-Het macrophages, the Rhodamine signal was reduced, but still detectable, while in *Ptpn2*-KO cells, no Rhodamine bright bacteria were visible, indicating that there was either a defect in bacterial transport to the lysosomes or a defect in lysosomal acidification (figure 5B). Consistent with this, staining with lysoTracker, which stains acidic lysosomes, was significantly reduced in *Ptpn2-*Het and *Ptpn2-*KO macrophages (figure 5C). To further investigate the mechanism leading to defective lysosomal acidification in *Ptpn2*-KO macrophages, we assessed the mRNA expression of proteins involved in transporting protein cargo from the endoplasmatic reticulum to lysosomes, or in maintaining the proton gradient across the lysosomal membrane, including cation-independent and cation-dependent mannose-6-phosphate receptor (CI-M6pr and CD-M6pr, respectively), glucocerebrosidase (Gba), the chloride channel Clc7, LAMP1 and LAMP2, and lysosomal integral membrane protein 2 (encoded by *Scarb2*). We found significantly reduced expression of CI-M6pr in *Ptpn2*-Het and *Ptpn2*-KO macrophages, consistent with defects in trafficking of mannose-6-phosphate tagged enzymes required for lysosomal acidification (figure 5D).^36^ Additionally, LAMP1 staining demonstrated that bacteria did not colocalise with lysosomes in *Ptpn2*-KO macrophages (figure 5E), indicating defective bacterial handling and compromised transport to lysosomes.

### Defects in bacterial transport to lysosomes together with defects of lysosomal acidification in *Ptpn2-KO* macrophages

**Figure 6:**
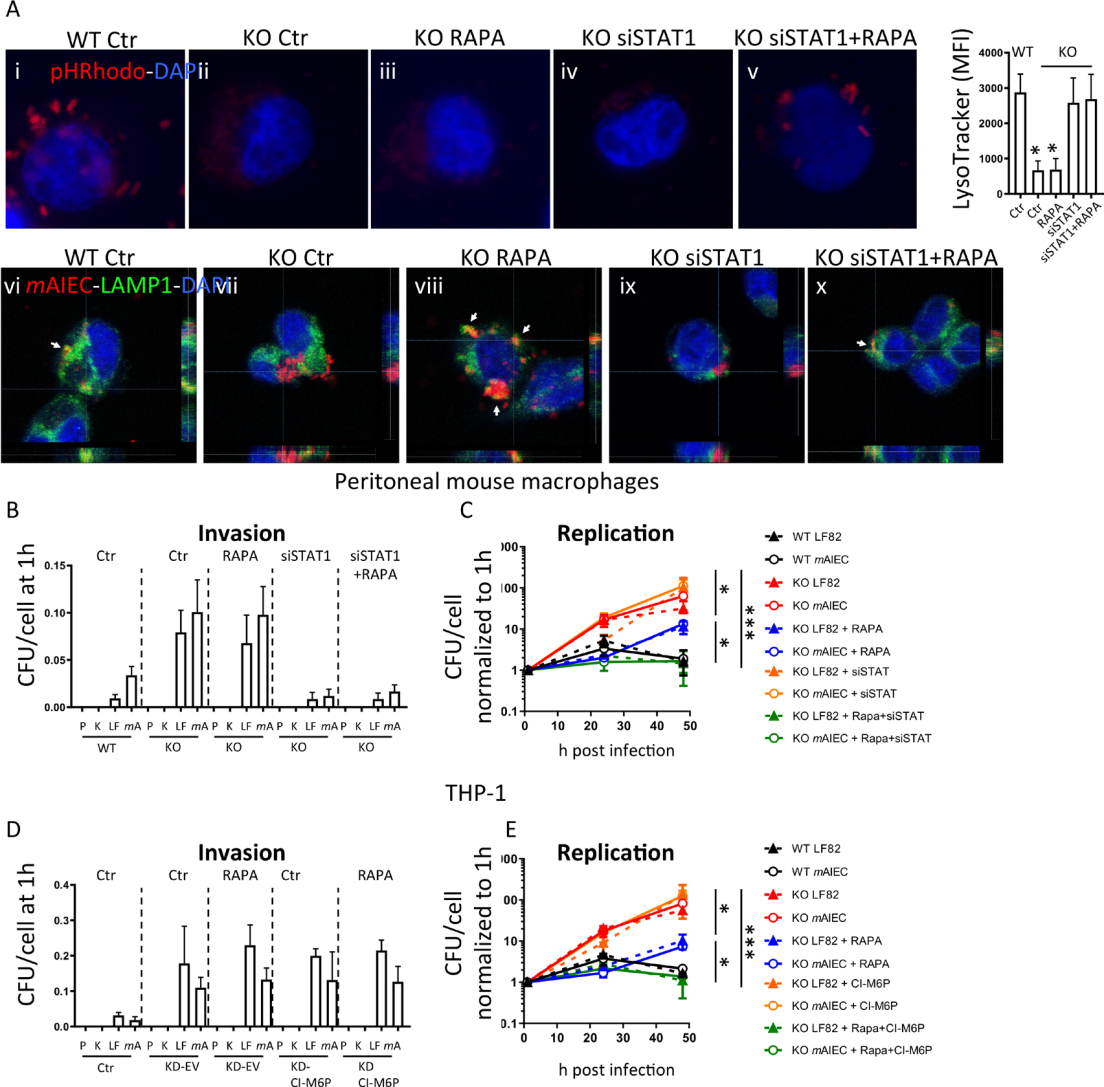
Autophagy activation combined with STAT1 silencing normalises bacterial handling in PTPN2-defective cells. (A) Peritoneal macrophages from WT, *Ptpn2-*Het (HET), or *Ptpn2*-KO (KO) mice were infected with pHRhodamine linked K12 (A, images in upper panel) or with mCherry-expressing *m*AIEC and autophagy induced using rapamycin and/or STAT1 silenced using siRNA. Depicted are representative fluorescent images and mean fluorescent intensity for LysoTracker stained cells. Scale bars: 10 µm. (B, C) Peritoneal macrophages were infected with K12 (K), LF82 (LF) or *m*AIEC (*m*A) and treated with rapamycin and/or STAT1 siRNA and analysed for (B) bacterial invasion after 1 hour, and (C) bacterial replication at the indicated time points. (D, E) THP-1 cells expressing PTPN2-specific shRNA (KD) were transfected with an empty vector (EV) or a CI-M6P overexpressing vector and infected with K12 (K), LF82 (LF) or *m*AIEC (*m*A) and treated with rapamycin. The graphs show (B) bacterial uptake after 1 hour, and (C) bacterial replication at the indicated time points. *P<0.05, **p<0.01, ***p<0.001, ANOVA. ANOVA, analysis of variance; *m*AIEC, mouse adherent-invasive *Escherichia coli*; M6P, mannose-6-phosphate; PTPN2, protein tyrosine phosphatase non-receptor type 2; STAT1, signal-transducer and activator of transcription.

Since autophagy is essential for intracellular transport of invading bacteria into lysosomes,^37^ we next addressed whether autophagy activation by rapamycin restores localisation of bacteria into lysosomes in *Ptpn2*-KO cells. In rapamycin-treated *Ptpn2*-KO macrophages, localisation of bacteria into lysosomes was indeed restored (figure 6A, colocalisation of *m*AIEC with LAMP1, subpanel viii), but there were still no pHRhodamine bright spots detectable on infection with pHRhodo particles, indicating that autophagy activation can restore the ability to transport bacteria into lysosomes in *Ptpn2*-KO cells, but that a defect in lysosomal acidification persists (figure 6A, subpanel iii). Treatment with IFN-γ and activation of STAT1 decreases expression of CI-M6PR.^38^ Thus, we hypothesised that the elevated levels of STAT1 observed in *Ptpn2*-KO macrophages might contribute to the reduced CI-M6pr protein expression and subsequent defect in lysosomal acidification in these cells. To test this hypothesis, we silenced STAT1 and subsequently activated autophagy by rapamycin treatment after infection with *m*AIEC or pHRhodo particles. In this setting, we clearly observed bacteria in LAMP1-positive lysosomes and pHRhodamine-bright spots (figure 6A, subpanels v and x). In addition to restoring lysosomal acidification in *Ptpn2-*KO macrophages, STAT1 silencing also restored CI-6Mpr expression (online supplemental figure 4). Concomitant STAT1 silencing and autophagy induction completely normalised the increased bacterial uptake and replication (figure 6B, C). Similar effects on bacterial replication were observed in rapamycin-treated PTPN2-deficient THP-1 cells that overexpressed CI-M6pr (figure 6D, E, online supplemental figure 4C). Taken together, this indicates that the defect in bacterial handling observed in *Ptpn2*-deficient cells results from a defect in both autophagy and lysosomal acidification, while restoration of both processes re-established the ability to clear intracellular bacteria in *Ptpn2*-KO cells.

### Mice lacking *Ptpn2* in macrophages are more susceptible to *m*AIEC infection

**Figure 7:**
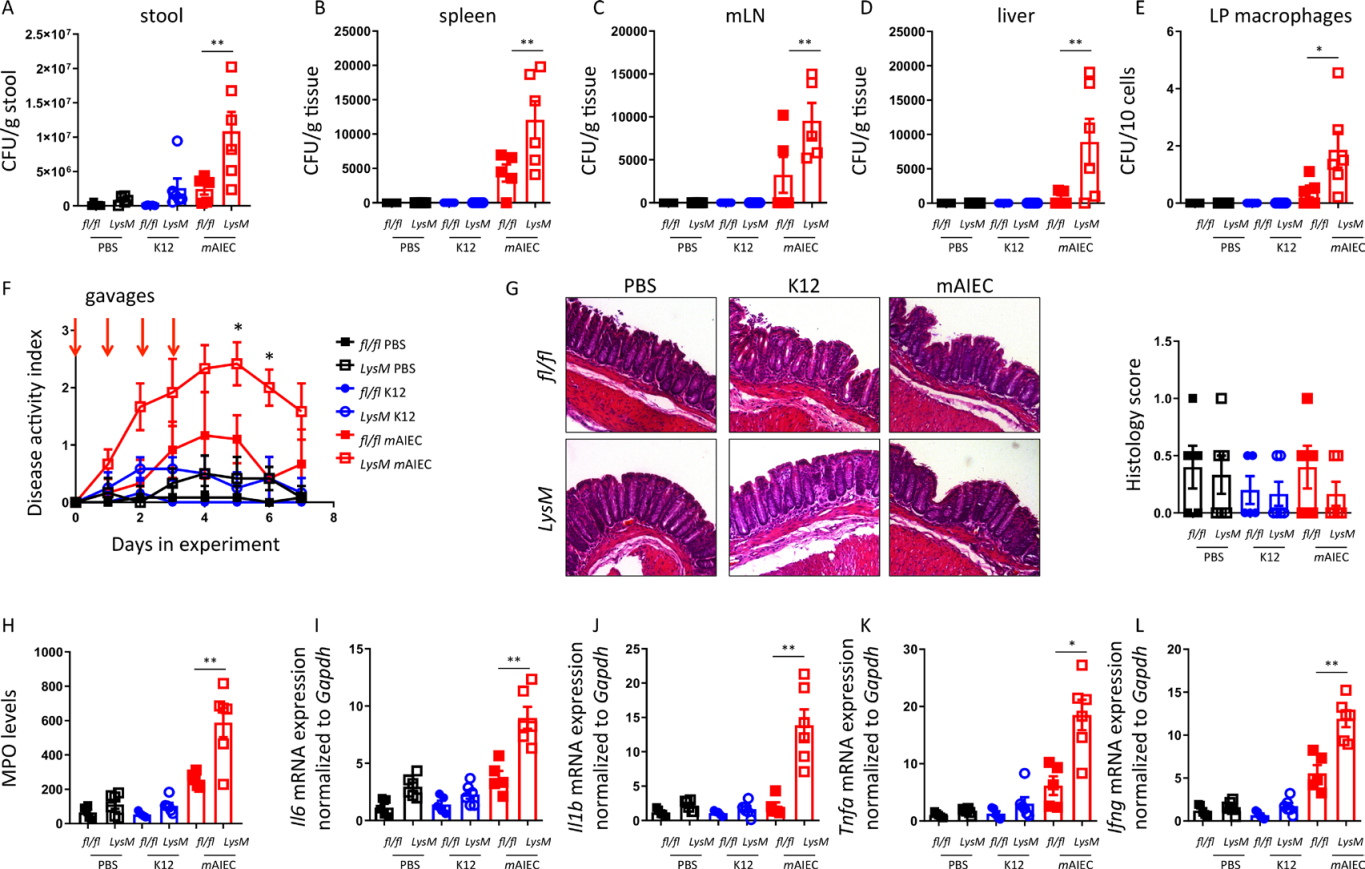
Mice lacking PTPN2 in macrophages are more susceptible to mAIEC infection.*Ptpn2^fl/fl^* and *Ptpn2*-LysMCre littermates were orally infected for 4 days with 10^9^ LF82 or *m*AIEC and *Escherichia coli* load in (A) the stool at day 5, (B) in the spleen, (C) mesenteric lymph nodes, (D) the liver and (E) lamina propria macrophages determined by plating on LB agar plates. (F) Disease activity index (DAI) over the course of the experiment. (G) Representative histological pictures and histopathology scoring. (H) Myeloperoxidase (MPO) levels in colon pieces. (I–L) mRNA expression levels of the indicated genes in the colon normalised to *Gapdh* and the median of untreated controls. *P<0.05, **p<0.01, ANOVA. ANOVA, analysis of variance; mAIEC, mouse adherent-invasive *Escherichia coli*; PTPN2, protein tyrosine phosphatase non-receptor type 2.

To test the in vivo consequences of defective bacterial handling in macrophages with PTPN2-loss, mice lacking PTPN2 in myeloid cells (*Ptpn2*-LysMCre mice; predominant PTPN2-loss in macrophages^26^) and *Ptpn2^fl^* ^/*fl*^ controls were orally gavaged on four consecutive days with 10^9^ *m*AIEC or non-invasive K12 *E. coli*. Consistent with an in vitro defect in bacterial handling, we observed increased *m*AIEC load in the stool of *Ptpn2*-LysMCre mice and enhanced bacterial translocation to the spleen, mesenteric lymph nodes and liver, and elevated bacterial counts in lamina propria macrophages (figure 7A–D). *mAIEC* induced mild disease only in *Ptpn2*-LysMCre but not *Ptpn2^fl^* ^/*fl*^ littermates (figure 7E, F), but there were no overt differences detectable in histological scores from infected vs non-infected and *Ptpn2^fl^* ^/*fl*^ or *Ptpn2*-LysMCre mice (figure 7G), while inflammatory markers, such as myeloperoxidase and mRNA levels of inflammatory cytokines were elevated in AIEC-infected *Ptpn2*-LysMCre mice (figure 7H–L). Consistent with our in vitro observations, activation of autophagy using rapamycin, or inhibition of CEACAM1 using an inhibitory anti-CEACAM1 antibody, partially prevented increased bacterial translocation/uptake in macrophages and normalised disease activity in *Ptpn2*-LysMCre mice (online supplemental figure 5). These data clearly indicate that loss of *Ptpn2* in macrophages hampers the clearance of potentially pathogenic bacteria and promotes susceptibility to *AIEC*-induced disease in vivo.

## Discussion

We demonstrate that normal *PTPN2* expression and function in intestinal macrophages/myeloid cells is crucial to clear intestinal adherent-invasive bacteria and that *PTPN2*-deficiency severely compromises effective macrophage-mediated elimination of invading bacteria, thus promoting AIEC*-*induced intestinal disease. Macrophages prevent systemic dissemination of bacteria that breach the epithelial barrier^39^ and macrophage-induced immune reactions towards luminal and adherent bacteria restrict overgrowth of pathogens and pathobionts,^40^ thus shaping the intestinal microbiota composition.^41–43^ The microbiome of patients with IBD carrying *PTPN2-*SNP rs1893217 differs from the microbiome of patients without the variant.^24 44^ In line with this, presence of SNP rs1893217 or loss of *Ptpn2* resulted in increased translocation of invasive bacteria, coupled with defective clearance, effects that might contribute to the changes in bacterial composition observed in *PTPN2* variant carrier IBD patients. The defective response to AIEC in macrophages with compromised *PTPN2* function is of clear clinical importance given the elevated levels of AIEC found in intestinal biopsies from IBD patients.^5^ In addition to explaining the alteration in microbial composition in *PTPN2* variant carriers, our findings identify additional mechanisms how *PTPN2* variants contribute to an increased risk of developing IBD.

*Ptpn2*-LysMCre mice lacking *Ptpn2* primarily in macrophages/monocytes^26^ showed increased adherence and invasion of AIEC on challenge, and furthermore show extra-intestinal translocation and AIEC-induced disease. Hence our data indicate that the defects in autophagy and lysosomal acidification observed in *Ptpn2*-deficient macrophages have a clear in vivo relevance.

Bacteria are actively taken up and subsequently degraded in macrophages by two distinct mechanisms: (1) uptake via endocytosis/phagocytosis and delivery into lysosomes,^45^ and (2) autophagy/xenophagy of bacteria, which targets bacteria that adhere to the membrane and enter the cytosol and/or escape the endosome-lysosome pathway.^33^ Intracellular vesicles are highly dynamic and interconnected, and autophagy vesicles promote the fusion of late endosomes with lysosomes.^34 35^ Thus, defects in autophagy, as observed in *PTPN2*-deficient/defective cells, crucially hamper the ability to combat invasive bacteria. This aligns with our observations and previously published in vitro studies showing defective autophagy in IEC and monocytes lacking *PTPN2*.^22^ Genetic studies point towards an important contribution of defective autophagy to the development of IBD,^46^ and variants in the autophagy-inducing receptor NOD2 and the autophagy-initiator molecule ATG16L1 were among the first genes associated with IBD.^47 48^ Bacterial products induce recruitment of autophagosomes to the site of bacterial entrance.^37^ It has been described that AIEC can subvert autophagy by blocking autophagosome-lysosome fusion, resulting in apoptosis of infected neutrophils and increased overall AIEC burden.^49^ These findings show that defects in autophagy hamper clearance of AIEC and ultimately promote survival of invading pathogens. Notably, we observed autophagy-activation not only in AIEC-infected cells, but also in the presence of non-invasive K12 *E. coli,* which was again abrogated in PTPN2-deficient macrophages. This indicates that loss of PTPN2 not only affects the response to pathogens/pathobionts, but also compromises physiological host reactions to benign commensals.

One important observation with regards to mouse models of IBD and studying the relevance of AIEC infections in those models is that the most widely used AIEC model strain, LF82, enters enterocytes and macrophages via binding to CEACAM6.^30^ However, mouse cells do not express CEACAM6 and are thus not very susceptible to infection with human AIEC strains.^30^ This issue is overcome in some studies by utilising transgenic mice that express human CEACAM3, 5, 6 and 7.^50^ The expression pattern of CEACAMs in IECs of these mice corresponds to that observed in humans, however, it also resulted in crypt hyperplasia and aberrant crypt morphology.^50^ Therefore, this model might not be optimally suited to study AIEC-host interactions. In contrast, our recently identified *m*AIEC^27^ was effective in invading mouse macrophages since it binds to and enters host cells by using CEACAM1, which is highly expressed in mouse enterocytes and intestinal macrophages.^51^ Thus, this novel *m*AIEC strain might represent a more appropriate strain for studying the effect of AIEC colonisation in mouse models of IBD.

AIEC manipulate the host response to infection, that is, it has been reported that the AIEC strain LF82 promotes its survival in macrophages via suppression of nuclear factor (NF)-κB signalling,^52^ but more recent studies showed that intracellular AIEC survival on early infection depends on NF-κB activation,^53^ while AIEC attenuate the very same signalling pathway later on.^54^ Furthermore, AIEC are more resistant to lysosomal superoxides than non-invasive *E. coli* strains.^54^ AIEC are also more resistant to the acidic pH in lysosomes, allowing them to survive within macrophages without the need to escape phagosomes/endosomes.^55^ This is of great interest given the reduced lysosomal acidification in PTPN2-deficient macrophages. Hence, loss of functional *PTPN2* likely facilitates intracellular survival of AIEC in two ways: (1) compromising transport of bacteria into lysosomes and (2) restricting acidification within lysosomes, thus compromising the cell’s ability to degrade bacteria.

In conclusion, we show that the autoimmune risk gene *PTPN2* is involved in handling invasive pathogens and its loss compromises macrophages to clear pathobionts. We not only identified several molecular mechanisms contributing to defects in bacterial handling, but also demonstrate the importance of efficient bacterial clearance by macrophages to prevent intestinal inflammation on AIEC infection. This might not only functionally explain the alterations observed in the microbial composition in patients with IBD carrying *PTPN2* SNP rs1893217,^24 44^ but may also explain, at least in part, why patients carrying this variant are more susceptible to the development of IBD.

## Data Availability

All data relevant to the study are included in the article or uploaded as online supplemental information.
