# Supplementary material for: Autoimmune susceptibility gene PTPN2 is required for clearance of adherent-invasive Escherichia coli by integrating bacterial uptake and lysosomal defence
Source: Gut. 2021 Feb 9;71(1):89–99. doi: 10.1136/gutjnl-2020-323636 (PMC8666829; doi:10.1136/gutjnl-2020-323636)
Supplement: Supplementary data [file gutjnl-2020-323636supp001.pdf]

Supplementary material to

The autoimmune susceptibility gene *PTPN2* is required for clearance of adherent-invasive *E. coli* by integrating bacterial uptake and lysosomal defense

Marianne R. Spalinger[1], PhD; Ali Shawki[1], PhD; Pritha Chatterjee[1], MSc; Vinicius Canale[1], MSc; Alina N. Santos[1], Anica Sayoc-Becerra[1], PhD; Michael Scharl[2], MD; Michel L. Tremblay[3], PhD; James Borneman[4], PhD; Declan F. McCole[1], PhD

1Division of Biomedical Sciences, School of Medicine, University of California Riverside, Riverside, California; 2Department for Gastroenterology and Hepatology, University Hospital and University of Zurich, Zurich, Switzerland; 3Department of Biochemistry and Goodman Cancer Research Centre, McGill University, Montreal, Quebec, Canada; 4Department of Microbiology & Plant Pathology, University of California Riverside, Riverside, California

Supplementary Methods

Macrophages. Peripheral blood mononuclear cells (PBMCs) were isolated from healthy controls and IBD patients by density gradient centrifugation on a Ficoll layer (20,000rpm for 20 min. at room temperature), washed twice in ice cold PBS and frozen in FCS containing 10% DMSO. PBMCs were then thawed, washed twice with RPMI (Life Technologies) 10% FCS and CD14+ cells isolated using the Miltenyi $CD14^+$ cell isolation kit according to the manufacturer's instructions. Sorted $CD14^+$ cells were incubated with hM-CSF (Peprotech; 50 ng/ml) and hIL-4 (Peprotech, 250 IU/ml) for 5 days and resulting macrophages used for infection studies.

For differentiation of THP-1 cells into macrophages, 10^6 cells were pulsed for 3 h with 50 ng/ml PMA, washed in serum-free RPMI and incubated for 48 h as described previously[1].

Bone marrow macrophages were prepared as described[1]. In brief, bone marrow was isolated from femori and tibiae, strained through a 70μm nylon mesh and cells incubated in differentiation medium (RPMI containing 1% pen/strep, 1% glutamine, 1% Na-pyruvate, 10 % FCS and 20 % L929 supernatant) for 7 days. On day 4, half of the culture medium was replaced by fresh differentiation medium.

Immunofluorescence staining. For immunofluorescence staining, cells were fixed with 4% paraformaldehyde for 10 min at room temperature, washed in PBS and fixed with methanol for 10 min at -20°C. After washing three times with PBS, unspecific antibody binding was blocked by incubation with 10% normal goat serum in Tris-buffered saline with 0.01% Tween-20 (TBS-T) for 2 h at room temperature prior to incubation with anti-LAMP-1 (1:200) or anti-LC3B (1:200) were used as primary antibodies. After washing 3 x in PS-Tween, secondary antibody was applied for 1 h at room temperature, cells washed three times in PBS-Tween and slides mounted with DAPI containing ProlongGold anti-fade mounting medium (Thermo Fisher Scientific). Images were taken on a Leica DM5500B microscope with a DFC450C camera (Leica) or a SP5 confocal microscope (Leica) and processed using the Leica Application Suite AF3.

RNA isolation. Cells were washed twice with ice cold PBS, and lyzed in RLT buffer (Qiagen) containing 0.25mM DTT for RNA isolation or RIPA buffer (50 mM Tris-Cl, 150mM NaCl, 1% NP-40, 0.5% sodium deoxycholate, 0.1% SDS) for protein isolation. RNA lysates were processed using the RNeasy mini kit from Qiagen according to the manufacturer's instructions and RNA concentration estimated measuring absorbance at 260 and 280 nm. Protein lysates were sonicated on ice for 30 seconds, centrifuged at 13,000g for 10 min and protein containing supernatants transferred to fresh tubes and protein concentrations measured using a BCA kit.

Western blotting. For Western blot analyses, equal amount of protein were loaded onto polyacrylamide gels and separated by SDS-PAGE. Proteins were blotted onto PVDF membranes, blocked in 3% milk, 1% BSA in TBS-T (Tris-buffered saline with 0.01% Tween20) prior to incubation with primary antibody overnight. Membranes were then washed three times in TBS-T, incubated with HRP-labeled secondary antibody for 1 h at room temperature, washed 3x with TBS-T and immunoreactive proteins visualized using an enhanced chemiluminescence kit (Thermo Fisher Scientific) and x-ray films (GE Healthcare Systems).

Quantitative PCR. Complementary DNA (cDNA) synthesis was performed using the qScript cDNA synthesis kit from Quantabio (Beverly, MA) following the manufacturer's instructions. Real-time PCR was performed using iQ SYBR Green Supermix (Bio-Rad, Hercules, CA) on a C1000 Thermal cycler equipped with a CFX96 Real-Time PCR system using BioRad CFX Manager 3.1 Software. Measurements were performed in triplicates, mouse GAPDH was used as endogenous control, and results were analyzed by the ΔΔCT method. The real-time PCR contained an initial enzyme activation step (3 min, 95 °C) followed by 45 cycles consisting of a denaturing (95 °C, 10 seconds), an annealing (53°-60°C, 10 seconds) and an extending (72 °C, 10 seconds) step. The used primers are listed in the Key Resources Table.

1 Spalinger MR, Kasper S, Gottier C, Lang S, Atrott K, Vavricka SR*, et al.* NLRP3 tyrosine phosphorylation is controlled by protein tyrosine phosphatase PTPN22. The Journal of Clinical Investigation 2016;**126.**

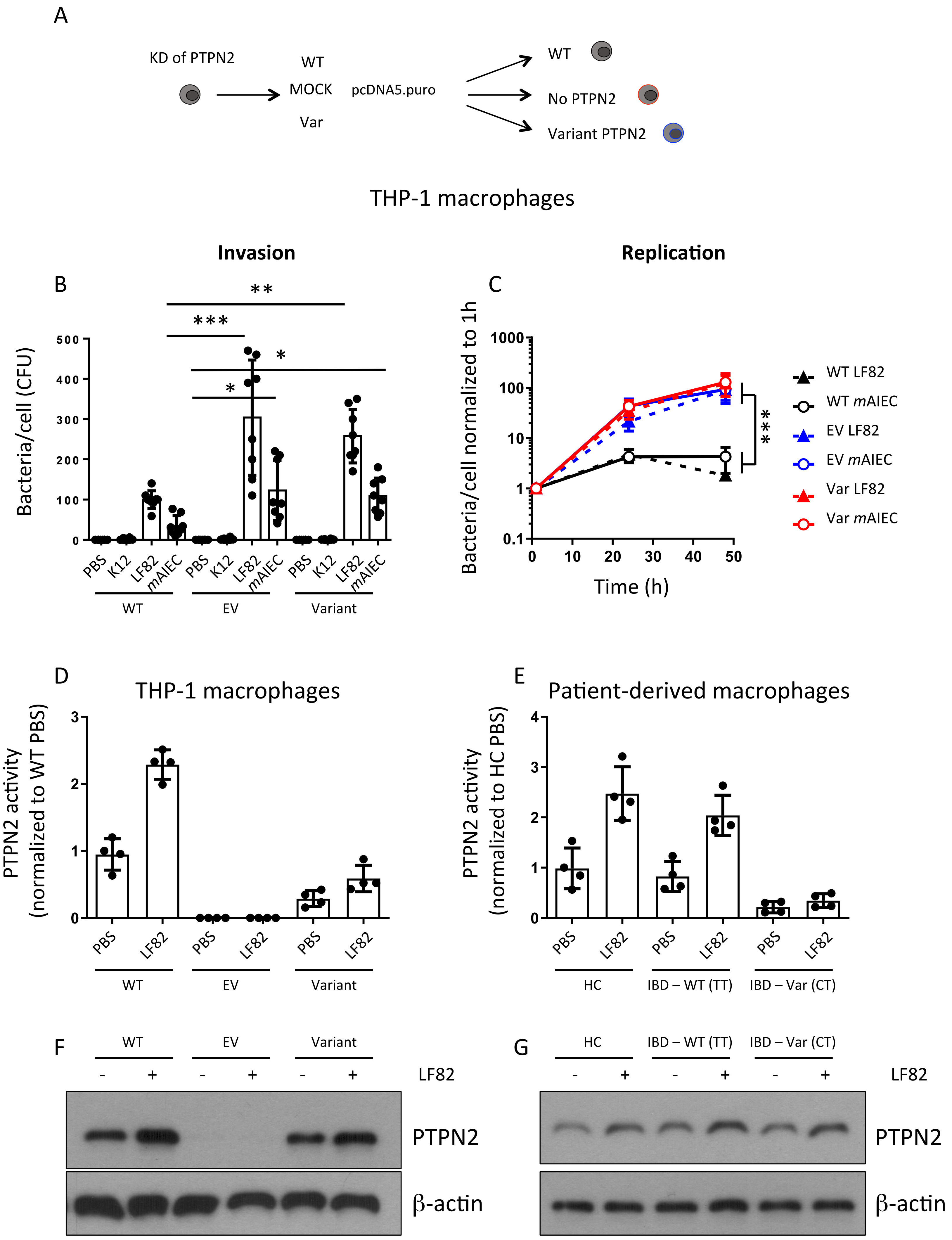

Supplementary Figure 1. Loss of PTPN2 promotes uptake and replication of AIEC in macrophages. *PTPN2* was knocked down in THP-1 cells prior to transfection with lentiviral particles containing an empty vector (EV), WT PTPN2 (WT) or Variant PTPN2 (Variant) as described in [22]. **A)** Schematic overview of the used cells; **B)** the cells were infected with non-invasive K12 *E. coli* or the AIEC strain LF82 for 2 h, washed with PBS and incubated with gentamycin and macrophage uptake analyzed after 1h. **C)** Bacterial replication at the indicated time-points in cells treated as in B). **D-G)** THP-1 cells (D+F) or patient derived macrophages (E +G) were infected with LF82 and analyzed for PTPN2 activity (D+E) and PTPN2 protein expression (F+G). *=p<0.05, **=p<0.01, ***=p<0.001, ANOVA. Related to Figure 1.

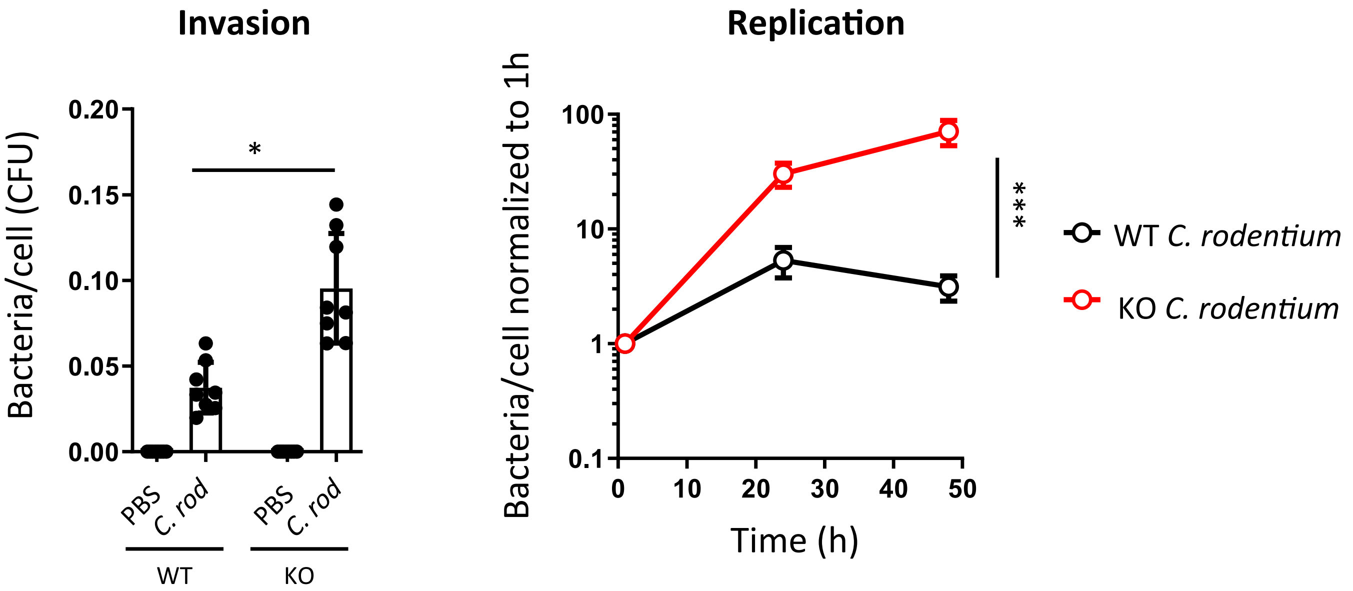

Supplementary Figure 2. Loss of PTPN2 promotes uptake and replication of C. *rodentium* in macrophages. Peritoneal macrophages from WT, and *Ptpn2*-KO (KO) mice were infected with *C. rodentium* and analyzed for **A)** bacterial uptake after 1h, and **B)** bacterial replication at the indicated time points. *=p<0.05, ***=p<0.001, ANOVA. Related to Figure 1.

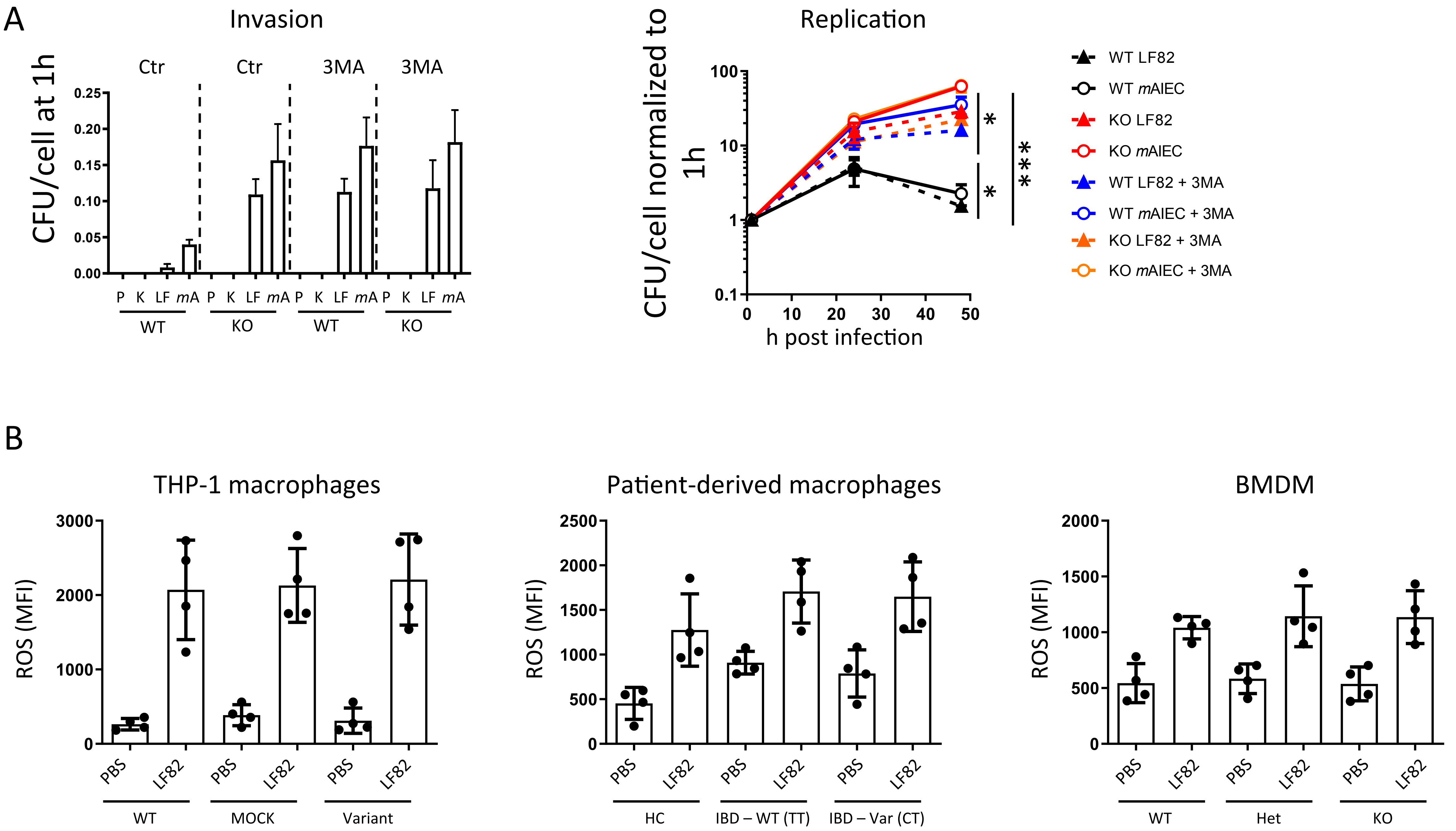

Supplementary Figure 3. Suppression of autophagy promotes bacterial replication in WT macrophages. A) Peritoneal macrophages from WT, and *Ptpn2*-KO (KO) mice were incubated for 1 h with 3-Methyladenine (3-MA) prior to infection with *m*AIEC and analysis of bacterial uptake after 1h and bacterial replication at the indicated time. P: PBS, K: K12, LF: LF82, *m*A: *m*AIEC. **B)** *PTPN2*-knockdown THP-1 cells expressing WT PTPN2, an empty vector (EV) or Variant PTPN2; monocyte derived macrophages from healthy controls (HC) or IBD patients being WT (WT) or heterozygous carriers of the PTPN2 Variant (Var); and bone marrow derived macrophages (BMDM) from *Ptpn2*-WT (WT) *Ptpn2*-Het (HET), or *Ptpn2*-KO (KO) mice were infected with LF82 and ROS production measured by flow cytometry. *=p<0.05, ***=p<0.001, ANOVA. Related to Figure 4.

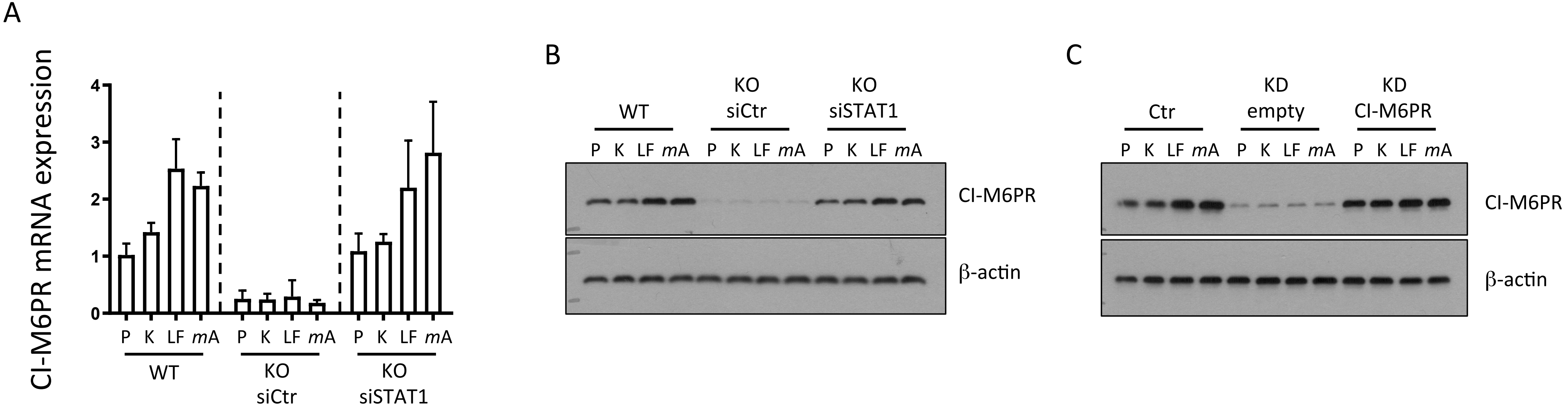

Supplementary Figure 4. STAT1 silencing restores CI-M6PR expression in *Ptpn2*-deficient macrophages. A+B: BMDM from *Ptpn2*-WT (WT) *and Ptpn2*-KO (KO) mice were treated with STAT1 siRNA for 24 h prior to infection with K12 (K), LF82 (LF) or *m*AIEC (*m*A) and analyzed for CI-M6PR **A)** mRNA and **B)** protein expression. **C)** THP-1 cells expressing PTPN2-specific shRNA were transfected with an empty vector (empty) or a CI-M6PR overexpressing vector prior to infection with K12 (K), LF82 (LF) or *m*AIEC (*m*A) and analyzed for CI-M6PR protein expression. P: PBS, K: K12, LF: LF82, *m*A: *m*AIEC. Related to Figure 6.

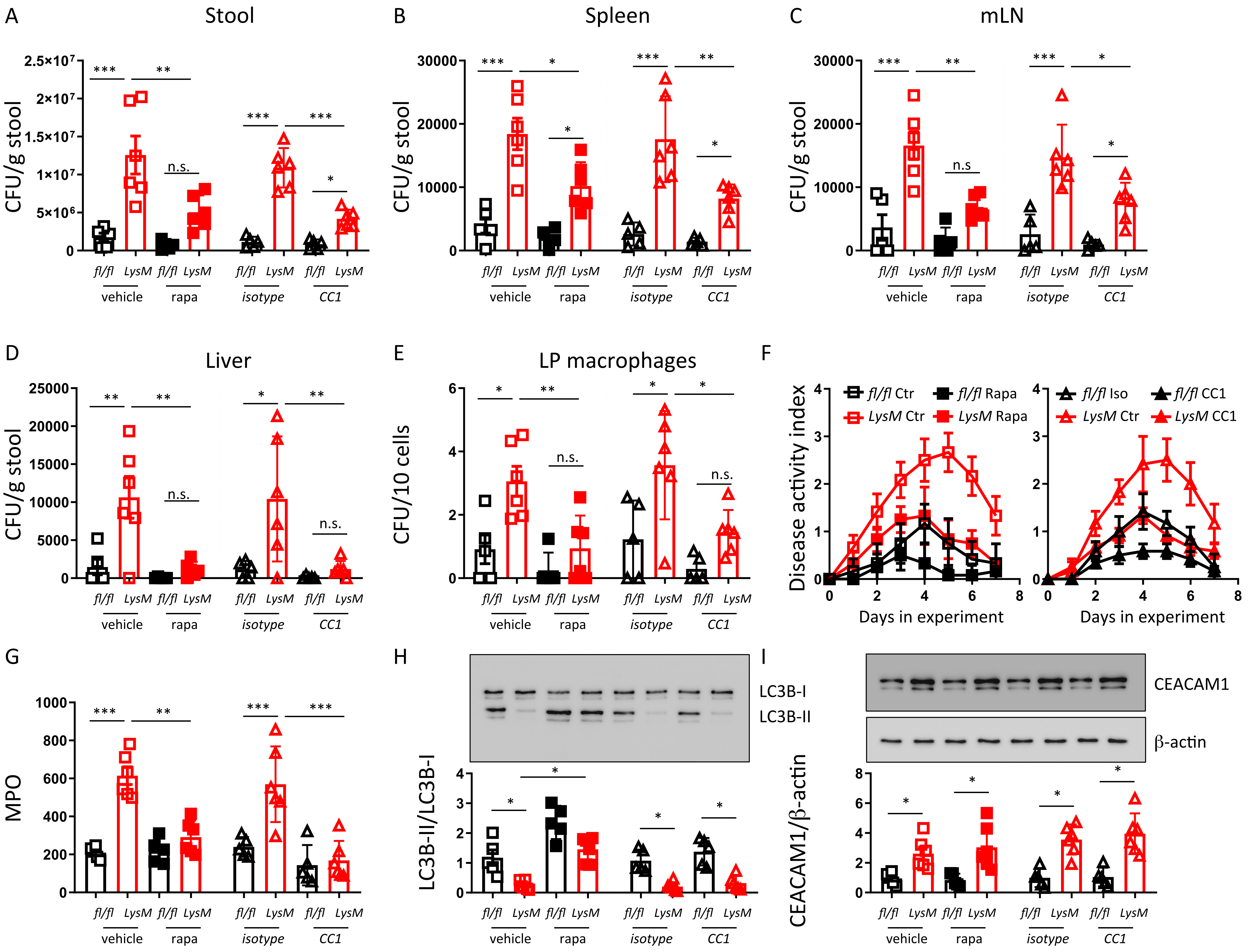

Supplementary Figure 5. Autophagy induction and CEACAM-1 inhibition partially rescue the increased susceptibility to *m*AIEC in *Ptpn2*-LysMCre mice. *Ptpn2*$^{fl/fl}$ and *Ptpn2*-LysMCre littermates were orally infected for 4 days with 10^9 *m*AIEC and treated daily with vehicle or rapamycin (rapa), or with an isotype control (isotype) or a CEACAM-1 blocking antibody (CC1) and *E. coli* load in **A)** the stool at day 5, **B)** in the spleen, **C)** mesenteric lymph nodes, **D)** the liver, and **E)** lamina propria macrophages determined by plating on LB agar plates. **F)** Disease activity index (DAI) over the course. **G)** Myeloperoxidase levels. Representative pictures and densitometry of **H)** LC3B-II normalized to LC3B-I, and **I)** CEACAM-1 normalized to b-actin. *=p<0.05, **=p<0.01, ***=p<0.001, ANOVA.

**REAGENT**	**SOURCE**	**Catalogue number or Reference**
**Antibodies**		
CEACAM6 mouse monoclonal (9A6)	Abcam	Ab78029
CEACAM1 rabbit monoclonal (D1P4T)	Cell signaling	14771
Rabbit anti p-STAT1 rabbit monoclonal (58D6)	Cell signaling	9167
Rabbit anti total STAT1 rabbit monoclonal (42H3)	Cell signaling	9175
TCPTP mouse monoclonal (F4-1D)	EMD Millipore	PH03L-100UL
β-actin rabbit monoclonal (D6A8)	Cell signaling	8457
ATG16L1 (rabbit monoclonal (D6D5)	Cell signaling	8089
p62 (mouse and human)		
LC3B mouse monoclonal (E5Q2K)	Cell signaling	83506
LAMP1 Rat monoclonal (1D4B)	Invitrogen	14-1071-82
M6PR-CI rabbit monoclonal (EPR6599)	Abcam	Ab124767
HRP-conjugated anti-mouse	Jackson Immunosearch	115-036-062
HRP-conjugated anti-rabbit	Jackson Immunosearch	111-036-045
AlexaFluor488-conjugated anti-rat	Jackson Immunosearch	712-546-153
**Bacterial and Virus Strains**		
E.Coli-K12	ATCC	25404
E.Coli-*m*AIEC	Own lab	Published in (Shawki et al., 2019)
E.Coli-LF82	A. Darfeuille-Michaud/Nicholas Barnich, Université d'Auvergne, Clermont-Ferrand, France	Published in (Darfeuille-Michaud et al., 2004)
**Biological Samples**		
Human patient derived macrophages	M. Scharl, University Hospital Zurich	Published in (Niechcial et al., 2020) Supplementary Table S2 for details
**Chemicals, Peptides, and Recombinant Proteins**		
Rapamycin	EMD Millipore	553210-100UG
iQ SYBR Green Supermix	Bio-Rad	1708882
Lutheria-Bertani medium		
Agarose		
Ficoll	GE Healthcare Life Sciences	17144002
DMSO	Dimethyl sulfoxide	276855-100ML
hM-CSF	PeproTech	300-25
hIL-4	PeprTech	200-04
Phorbol 12-Myristate 13-Acetat (PMA)	Sigma-Aldrich	P8139-5MG
RPMI 1640	Corning	10-040-CV
Gentamicin	Sigma-Aldrich	G1397-10ML
PVDF membranes	EMD Millipore	IPVH00010
Bovine serum albumin (BSA)	Sigma-Aldrich	A9418-100G
**Commercial Assays**		
pHrodo™ Red E. coli BioParticles™ Conjugate for Phagocytosis	Thermo Fisher Scientific	P35361

CD14 MicroBeads, human	Miltenyi Biotec	130-050-201
RNeasy Mini Kit	Qiagen	74106
Thermo Scientific Pierce BCA Protein Assay	Thermo Fisher Scientific	23228
SuperSignal™ West Pico PLUS Chemiluminescent Substrate	Thermo Fisher Scientific	34577
qScript cDNA Synthesis Kit	Quantabio	95074-100
**Cell Lines**		
THP-1 MOCK	Scharl lab, University Hospital Zurich	Published in (Scharl et al., 2012a)
THP-1 WT	Scharl lab, University Hospital Zurich	Published in (Scharl et al., 2012a)
THP-1 Var	Scharl lab, University Hospital Zurich	Published in (Scharl et al., 2012a)
NCTC clone 929 (L929 cells)	ATCC	ATCC® CCL-1™
THP-1 cells	ATCC	ATCC® TIB-202™
**Mice**		
Balb/c PTPN2 KO mice	M. L. Tremblay, McGill University	Published in (You-Ten et al., 1997)
PTPN2-LysMCre mice	Scharl lab, University Hospital Zurich	Published in (Spalinger et al., 2018)
**Oligonucleotides**		
STAT1 siRNA	Dharmacon	L-003543-00-0005
Non-targeting siRNA	Dharmacon	D-001810-10-05
Primers for qPCR	IDT	Details in supplementary Table S2
**Recombinant DNA**		
mCherry plasmid (pKB4985)	Lo Lab, University of Riverside	n/a

Supplementary Table 1: Materials used in this manuscript.

BMJ Publishing Group Limited (BMJ) disclaims all liability and responsibility arising from any reliance placed on this supplemental material which has been supplied by the author(s)

Patient #	Genotype	Sex	Age	Diagnosis	Medication
1	WT (TT)	M	63	Crohns Disease	Budenofalk, Humira
2	TG (CT)	M	35	Ulcerative Colitis	No disease specific medication
3	TG (CT)	M	40	Crohns Disease	Cimzia (Certolizumab pegol), Puri-Nethol, Budenofalk
4	WT (TT)	M	40	Crohns Disease	Colosan, Infliximab
5	WT (TT)	W	39	Ulcerative Colitis	Entyvio (Vedolizumab)
6	WT (TT)	W	47	Ulcerative Colitis	Entyvio (Vedolizumab)
7	TG (CT)	M	62	Crohn's Disease	Entyvio (Vedolizumab), Salofalk (Mesalazine)
8	TG (CT)	M	45	Ulcerative Colitis	Salofalk (Mesalazine)
9	WT (TT)	W	39	Ulcerative Colitis	Entyvio (Vedolizumab)
10	TG (CT)	W	42	Ulcerative Colitis	Entyvio (Vedolizumab)
11	WT (TT)	W	62	Crohn's Disease	Entyvio (Vedolizumab), Salofalk (Mesalazine)
12	WT (TT)	M	57	Crohn's Disease	Salofalk (Mesalazine)
13	TG (CT)	W	54	Crohn's Disease	Entyvio (Vedolizumab), Salofalk (Mesalazine)
14	TG (CT)	M	61	Crohn's Disease	Salofalk (Mesalazine)
15	WT (TT)	M	42	Crohn's Disease	Colosan, Infliximab
16	WT (TT)	W	52	Ulcerative Colitis	No disease specific medication
17	TG (CT)	W	44	Ulcerative Colitis	No disease specific medication
18	WT (TT)	W	39	Healthy control	n/a
19	WT (TT)	M	45	Healthy control	n/a
20	WT (TT)	M	40	Healthy control	n/a
21	TG (CT)	W	51	Healthy control	n/a
22	TG (CT)	W	39	Healthy control	n/a

Supplementary Table 2: Patient characteristics.
